# Association Between Electrocardiographic Left Atrial Enlargement and Echocardiographic Left Atrial Indices Among Hypertensive Subjects in a Tertiary Hospital in South South Nigeria

**DOI:** 10.7759/cureus.34330

**Published:** 2023-01-29

**Authors:** Henry O Aiwuyo, John O Osarenkhoe, Ejiroghene M Umuerri, Fredrick I Aigbe, Austine Obasohan

**Affiliations:** 1 Internal Medicine, Brookdale University Hospital Medical Center, New York, USA; 2 Internal Medicine/Cardiology, Igbinedion University Teaching Hospital, Benin City, NGA; 3 Internal Medicine/Cardiology, Delta State University Teaching Hospital, Oghara, NGA; 4 Medicine, College of Medical Sciences, University of Benin, Benin City, NGA

**Keywords:** left atrial linear diameter, left atrial volume, left atrial enlargement, echocardiograph, electrocardiograph

## Abstract

Introduction: Left atrial (LA) enlargement poses a clinically significant risk of adverse cardiovascular outcomes for patients. To maximize the utility of LA size in diagnosis, its accurate measurement using electrocardiogram (ECG) and echocardiogram (ECHO) to assess LA linear diameter and LA volumes is expedient. The LA volumes correlate better than LA linear diameter with diastolic function variables. It is therefore expedient to use LA volumes routinely in assessing LA size as they may detect early and subtle changes in LA size and function.

Methods: A descriptive cross-sectional study was conducted on 200 adult hypertensive patients attending the outpatient cardiology clinic at Delta State University Teaching Hospital, Oghara, Nigeria, irrespective of blood pressure control and duration of hypertension whether on antihypertensive medications or not. The SPSS version 22 (IBM Corp., Armonk, NY, USA) was used for data management and analysis.

Result: There was a significant association between electrocardiographic left atrial (ECG-LA) enlargement and echocardiographic left atrial (ECHO-LA) size (LA linear diameter and LA maximum volume) in the study. Logistic regression analysis showed a significant odds ratio for all associations. With LA linear diameter as standard for assessing LA enlargement, the ECG had a sensitivity of 19%, specificity of 92.4%, a positive predictive value of 51%, and a negative predictive value of 73% in detecting LA enlargement. Using ECHO-LA maximum volume as a standard for assessing LA enlargement, the ECG had a sensitivity of 57.3%, a specificity of 67.7%, a positive predictive value of 42.9%, and a negative predictive value of 79% in detecting LA enlargement. The LA maximum volume showed relatively higher sensitivity and negative predictive values while LA linear diameter showed relatively higher specificity and positive predictive values.

Conclusion: A good association exists between ECG-LA enlargement and ECHO-LA enlargement. However, in ruling out LA enlargement on ECG, it is better to use LA maximum volume as a standard rather than the LA linear diameter.

## Introduction

Studies have shown left atrial (LA) enlargement to be an independent predictor of cardiovascular outcomes including congestive heart failure, myocardial infarction, need for revascularization, stroke, and death; this has been more evident in the elderly population who have not suffered cardiovascular disease [1]. The ability of the left atrium to compensate adequately is argued to be a measure of the ischemic burden and also the extent of left ventricle (LV) diastolic dysfunction [2]. A study showed that in patients with isolated diastolic dysfunction, LA enlargement was significantly associated with lower exercise capacity [3]. The LA diameter assessment (M-mode) is less accurate than volume measurements. Various cardiovascular diseases and LA compression can cause asymmetry in LA diameter [4].

The left atrium is sensitive to changes in left ventricular filling pressure, which is expressed as LV diastolic dysfunction. The left atrium is a marker of not only the severity of LV diastolic function but also its chronicity [5]. Some studies have shown that LA volume is a marker of a different cardiovascular outcome [6,7].

Electrocardiography (ECG) can be used in determining the size of various heart chambers, including the left atrium. The accuracy of this tool in determining chamber size is not comparable to echocardiography (ECHO), which gives a real-time assessment of LA size as well as function. The sensitivity and specificity of the ECG are low compared to higher diagnostic modalities such as 2D echocardiography, CT, and cardiac MRI.

This study aims to determine whether an association exists between electrocardiographic left atrial (ECG-LA) enlargement and echocardiographic left atrial (ECHO-LA )enlargement. We also intend to determine the sensitivity and specificity of the ECG in relation to ECHO-measured LA linear diameter and LA maximum volume. The ECG is more readily available for quick assessment of electrical and structural conditions of the heart compared to ECHO.

This study was done in a low socio-economic area where access to imaging modalities is not always available and diagnosis can be delayed in such instances. The outcomes of this study will help strengthen preventive cardiology practice, especially in resource-poor settings. Getting to know if a relationship exists or not will help predict within a reasonable limit of accuracy and allow physicians to make a clinical judgment before patients are transferred to tertiary centers where they can access advanced imaging modalities. This study also serves to emphasize the need for prevention as untreated hypertension is a major risk factor for LV remodeling which can lead to a dilated left atrium and consequently predispose to atrial arrhythmias, especially atrial fibrillation. 

## Materials and methods

A descriptive cross-sectional study was conducted on 200 adult hypertensive patients attending the outpatient cardiology clinic at Delta State University Teaching Hospital, Oghara, Nigeria. Ethical approval was obtained from the Health Ethics and Research Committee of the Delta State University Teaching Hospital (approval no. DELSUTH/HREC/2014/007).

Inclusion & exclusion criteria

The inclusion criteria for the study include the following: adult males and females ≥18 years; hypertensive patients irrespective of blood pressure control, whether on antihypertensive medications or not; and duration of hypertension. The exclusion criteria for the study were as follows: patients with overt heart failure, cardiomyopathy, suboptimal echocardiographic images, rheumatic valvular heart disease (regurgitation or stenosis); non-consenting patients; patients with atrial fibrillation; and pregnant women.

Sampling technique

A total of 200 hypertensive patients were recruited from the cardiology clinics. A systematic sampling method was used to recruit these patients. An average of 30 hypertensive patients visit the cardiology clinics in a week. A total of 720 hypertensive patients are therefore expected to visit the clinic in six months. Using the formulae for estimating sampling interval= x/sample size (where x is the total study population) the sampling ratio (interval) was found to be 3:1. A sampling frame was then arranged which comprised 720 participants. Simple random sampling using the balloting method was used to identify the first participant, and every third participant was recruited for the study. The average time duration for a revisit to the clinic is two months and so participants who were booked for a revisit within a period of fewer than two months were not recruited for the study to avoid the problem of double entry.

A total of 100 apparently healthy controls without any known medical or cardiovascular disease, aged 18 years and above, and normotensive were recruited for the study. They were largely drawn from hospital workers, visitors, and patient relatives. Those with evidence of structural heart disease and sub-optimal echocardiographic images were excluded from the study. They were matched against the test subjects for age and sex in a 1:2 ratio.

Informed consent was obtained from participants and was duly signed/thumb printed before they were co-opted for the study. Confidentiality and the right to exit from the study at any time were maintained.

A transthoracic ECHO (Xario diagnostics ultrasound system model SSA-660A, Toshiba Medicals, Tochigi, Japan) with ECG gating was performed according to established recommendations [8]. The M-mode, 2D, spectral Doppler, and tissue Doppler echocardiographic images were obtained from standard echocardiographic views (parasternal, apical) with all subjects in the left lateral decubitus position.

The LA dimension was measured according to the American Society of Echocardiographyb (ASE) i.e., from the leading edge of the posterior aortic wall to the leading edge of the posterior wall of the left atrium at end-systole [8]. Adebayo et al. found a value of 3.1±0.47 (2.16 - 4.04cm) for LA linear diameter amongst normal healthy controls [9]. In the study, LA size changes were compared between hypertensive patients and normal healthy controls [9]. This value was used to define the normal LA linear dimension.

The LA maximum volume (just before mitral valve opening at the peak of the T-wave on ECG), LA minimal volume (at mitral valve closure at the QRS complex of the ECG), and LA pre ‘a’ wave volume (onset of the P wave on ECG) were measured using the biplane Modified Simpson’s method of discs. The views for this method were apical 4 and 2 chambers. Accurate tracings were made to avoid overestimation. The LA appendage and the pulmonary veins were not included in the tracings.

All volumes were indexed to body surface area. The LA enlargement is defined as a maximum LA indexed volume ≥29mL/m2 (normal = 16mL/m2 to 28mL/m2, mild enlargement = 29 to 33; moderate enlargement = 34mL/m2 to 39mL/m2, severe enlargement ≥40mL/m2), and LA volume (normal = 18mls to 58mls, mild enlargement = 59mls to 68mls, moderate enlargement = 69mls to 78mls, severe enlargement = ≥79mls) according to the ASE guidelines [8]. However, for this study, normal LA maximum volume was defined as mean LA maximum volume±2 standard deviation (SD) using the value from the control arm of the study which was 39.10±13.54mls. 

The LA enlargement on ECG was defined as the presence of P wave duration >0.12cm in lead II or increased duration and depth of the downward deflection of biphasic P wave in V1 so that the area subtended by it is >0.04mm-sec [10].

A semi-structured (containing both open- and closed-ended questions) interviewer-administered questionnaire was used for each participant recruited for the study and checked properly each day for completeness. Frequency distributions and two-way tables were used to summarize the data. Statistical Package for the Social Sciences (SPSS) version 22 (IBM Corp., Armonk, NY, USA) was used for data analysis. Continuous variables were calculated as mean ± SD while categorical data were expressed as frequency and percentages. Continuous and categorical variables between cases and controls were compared using unpaired samples Student’s t-tests and χ2 analyses, respectively. Differences in a two-tailed test p<0.05 was considered statistically significant. Specificity = True Negatives / True Negatives + False Positives, Sensitivity = True Positives / True Positives + False Negatives, Positive Predictive Value = True Positives / True Positives + False Positives, Negative Predictive Value = True Negatives / True Negatives + False Negatives.

## Results

There was a significant association between ECG-LA enlargement and ECHO-LA size (LA linear diameter and LA maximum volume) in the study. Logistic regression analysis also showed a significant odds ratio for all associations (Table 1).

**Table 1:** Association between ECG-LA enlargement and ECHO-LA enlargement #significant at  p≤0.05 +significant odds ratios at respective confidence intervals ECHO-LA: Echocardiographic left atrial, ECG-LA: Electrocardiographic left atrial, CI: Confidence interval, OR: Odds ratio, χ2: Chi-square test statistics

	ECHO-LA Enlargement	ECG-LA Enlargement		Total	χ2	p-value	OR	CI
		Yes	No					
ECHO-LA Linear Diameter	Enlarged	17 (51.5)	16 (48.5)	33 (100)	8.48	0.004^#^	2.88	1.30 - 6.37^+^
	Normal	72 (27.0)	195 (73.0)	267 (100)				
	ECHO-LA Maximum volume	Enlarged	51 (42.9)	68 (57.1)	119 (100)	16.45	<0.001^#^	2.82	1.64 - 4.86^+^
Normal		38 (21.0)	143 (79.0)	181 (100)				

The LA maximum volume showed relatively higher sensitivity and negative predictive values while LA linear diameter showed relatively higher specificity and positive predictive values (Tables 2, 3).

**Table 2:** Comparison of diagnostic accuracy between ECG-LA enlargement and ECHO-LA enlargement using LA linear diameter NPV: Negative predictive value, PPV: Positive predictive value, ECG: Electrocardiogram, ECGO: Echocardiogram, LA: Left atrial

Diagnostic Accuracy Parameter	ECG	ECHO
Sensitivity (%)	19.0	57.3
Specificity (%)	92.4	67.7
PPV (%)	51.0	42.9
NPV (%)	73.0	79.0

**Table 3:** Comparison of diagnostic accuracy between ECG-LA enlargement and ECHO-LA enlargement using LA maximum volume NPV: Negative predictive value, PPV: Positive predictive value, ECG: Electrocardiogram, ECGO: Echocardiogram, LA: Left atrial

Diagnostic Accuracy Parameter	ECG	ECHO
Sensitivity (%)	43.9	32.6
Specificity (%)	93.8	96.7
PPV (%)	92.1	98.9
NPV (%)	50.2	13.7

## Discussion

The ECG remains widely used for LA enlargement detection due to its simplicity and accessibility. However, ECHO has become the gold standard in clinical practice. In our study, the prevalence of LA enlargement in the hypertensive group was found to be 16% and 59% using LA linear diameter and LA maximum volume respectively. This prevalence is in keeping with a systematic review by Cuspidi et al. which found the prevalence of ECHO-LA enlargement to vary between 16% to 83%. The studies applied both LA linear diameter and LA maximum volume for LA size measurement [11]. Adewole et al. in a hospital-based study conducted in Ibadan, Nigeria, found the prevalence of LA enlargement among newly diagnosed hypertensive subjects to be 15.8% using LA linear diameter [12].

A study by van Dam et al. on ECG-ECHO study of LA enlargement among 100 adult patients, found that the left atrium was enlarged in 13 of the participants [13]. Only four patients met the ECG criteria for LA enlargement, and 20 patients showed echocardiographically proven LA enlargement. They stated that there was no relationship between the ECG diagnosis of LA enlargement and the LA size as measured by the ECHO [13].

Waggoner et al. showed from their study that the sensitivity and specificity of ECG-LA enlargement in detecting enlarged left atrium were 67% and 94.4%, respectively [14]. Left atrial enlargement by ECG and ECHO was seen in 307 patients in sinus rhythm. The overall predictive index of the electrocardiogram for LA enlargement using ECHO as the standard was found to be 63% and that for the absence of LA enlargement was 78%. They concluded that ECHO is better at diagnosing LA enlargement than the ECG criteria [14].

In a similar study by Waqas et al. on the sensitivity and specificity of ‘P’ mitrale on ECG in diagnosing LA enlargement on ECHO, they discovered that ‘P’ mitrale has a sensitivity of 22.5% and a specificity of 100% [15]. The positive predictive value was 100% whereas the negative predictive value was 24.39%. The diagnostic efficacy of the test was calculated as 38%. They, therefore, concluded that though the sensitivity of ECG is low in detecting LA enlargement, its sensitivity can be increased by combining ‘P’ terminal force in lead V1 and ‘P’ mitrale as an additional criterion in lead 2. Lee et al. showed an association between ECG-LA enlargement and ECHO-LA volume enlargement [10]. They used the modified Simpson’s method of disks in estimating the LA volume on ECHO and found the biphasic “P” wave in lead V1 to be specific and the “P” wave duration in lead 2 to be sensitive in detecting ECHO-LA enlargement. They also recommended that “P” wave abnormalities should be regarded as LA abnormalities and not LA enlargement due to the low sensitivity of ECG in detecting ECHO-LA enlargement [10].

Phasic LA volumes (maximum, minimum, pre A) were shown to be higher among the hypertensive subjects, and these findings have been consistently demonstrated in prospective studies and found to be more reliable in predicting cardiovascular outcomes than the LA linear diameter [16,17]. The LA diameter assessment (M-mode) is less accurate than volume measurements; various cardiovascular diseases and compression on the left atrium can cause asymmetry in LA diameter. This may suggest that LA volume measurement may be a better way to assess LA function among hypertensive patients on treatment.

This study showed a significant association between ECG-LA enlargement and ECHO-LA (maximum volume/linear diameter) enlargement among normotensive controls and hypertensive patients. An association has been shown to exist between ECG and ECHO-LA enlargement; however, the ECG criterion for LA enlargement has poor sensitivity for predicting LA enlargement on ECHO [10]. In our opinion, the reasons for poor sensitivity could be intrinsic to the modality of the assessment itself as the ECG utilizes graphical electrical signals to define structural heart problems making it a poor discriminator of the overall size of the LA chamber. The body habitus of patients can also affect the ECG to a larger degree than ECHO, thus limiting its sensitivity to identify LA enlargement if present. 

Waggoner et al. in their study correlating ECG and ECHO-LA enlargement showed that the ECG had good specificity but poor sensitivity in detecting LA enlargement [14]. This study used the two criteria stated earlier as a case definition for ECG-LAE. Although the ECG had low sensitivities and negative predictive values in detecting ECHO-LA enlargement, LA volume was relatively better than LA linear diameter with regard to sensitivity and negative predictive values.

In our study, the specificity and positive predictive values were higher when LA linear diameter was used than with LA maximum volume. Therefore, the false negative rates were lower when LA volume was used as the standard test than with LA linear diameter, making it a better “rule out” test than LA linear diameter. The LA linear diameter had a lower false positive rate than the LA volume making it a better rule in a test. This finding is in keeping with the study by Waqas et al [15]; however, more studies will need to be done to clearly buttress these findings.

Limitations

This is a hospital-based study of a selected group limiting the generalization of its findings. Some of the patients recruited have been on anti-hypertensive medications prior to the study and so it may be difficult to exclude any influence of prior treatment. We also did not factor in the duration of hypertension in our work as some of our patients may have been chronically hypertensive.

A detailed history and review of blood pressure were recorded, however, it was beyond the scope of this study to perform 24-hour ambulatory blood pressure measurements as some of the patients in the control arm may have had masked hypertension. Although patients with atrial fibrillation (AF) were excluded from the study, the occurrence of paroxysmal AF may not be entirely excluded, as Holter monitoring was not performed.

Recommendations

Follow-up studies in the long term are required to determine the prognosis of LA enlargement which may also factor in the duration of hypertension or use clusters of newly diagnosed hypertensive patients versus controls. We should not rely on the ECG as a standard for diagnosing LA enlargement. It is necessary to use ECHO as a standard due to the poor sensitivity of ECG-LA enlargement. The LA volume assessment should be done routinely using ECHO especially when LA linear diameter is normal as it may be essential for risk stratification.

## Conclusions

This study demonstrated that a strong association exists between ECG-LA enlargement and ECHO-LA enlargement. However, in ruling out LA enlargement on ECG, it is better to use LA maximum volume as a standard rather than LA linear diameter. Left atrial enlargement on ECG can better be assessed using a combination of P terminal force on lead V1 and P mitrale on lead 2.
